# A Survey on the Effect That Medical Cannabis Has on Prescription Opioid Medication Usage for the Treatment of Chronic Pain at Three Medical Cannabis Practice Sites

**DOI:** 10.7759/cureus.11848

**Published:** 2020-12-02

**Authors:** Kevin M Takakuwa, Dustin Sulak

**Affiliations:** 1 Emergency Medicine, Society of Cannabis Clinicians, Sebastopol, USA; 2 General Practice, Integr8 Health, Falmouth, USA

**Keywords:** medical cannabis, marijuana, opioid use, chronic pain, survey

## Abstract

Objective: The opioid epidemic continues to claim thousands of lives every year without an effective strategy useful in mitigating mortality. The use of medical cannabis has been proposed as a potential strategy to decrease opioid usage. The objective of this study was to determine how the use of medical cannabis affects prescribed opioid usage in chronic pain patients.

Methods: We conducted an online convenience sample survey of patients from three medical cannabis practice sites who had reported using opioids. A total of 1181 patients responded, 656 were excluded for not using medical cannabis in combination with opioid use or not meeting the definition of chronic pain, leaving 525 patients who had used prescription opioid medications continuously for at least three months to treat chronic pain and were using medical cannabis in combination with their prescribed opioid use.

Results: Overall, 40.4% (n=204) reported that they stopped all opioids, 45.2% (n=228) reported some decrease in their opioid usage, 13.3% (n=67) reported no change in opioid usage, and 1.1% (n=6) reported an increase in opioid usage. The majority (65.3%, n=299) reported that they sustained the opioid change for over a year. Almost half (48.2%, n=241) reported a 40-100% decrease in pain while 8.6% (n=43) had no change in pain and 2.6% (n=13) had worsening pain. The majority reported improved ability to function (80.0%, n=420) and improved quality of life (87.0%, n=457) with medical cannabis. The majority (62.8%, n=323) did not want to take opioids in the future. While the change in pain level was not affected by age and gender, the younger age group had improved ability to function compared with the middle and older age groups.

Conclusions: Patients in this study reported that cannabis was a useful adjunct and substitute for prescription opioids in treating their chronic pain and had the added benefit of improving the ability to function and quality of life.

## Introduction

The US opioid epidemic continues unabated with an estimated 10.3 million people misusing opioids in 2018, representing 3.7% of the population [1] and accounting for 47,600 overdose deaths in 2017 [2]. The use of cannabis in conjunction with opioids has been proposed as a strategy to manage opioid withdrawal or dependence since the mechanisms of the endocannabinoid system may work in tandem with the opioid system [3-7]. Human subjects [8,9] and animal studies [10-11] seem to support the hypothesis that the addition of cannabis to opioids reduces pain, perhaps synergistically, and could lead to decreased doses or even cessation of opioids [12].

These findings have been reported in studies showing reduced opioid doses with the addition of cannabis [13-16] and reduced pain when cannabis is added to opioid use [14,16,17]. Observational studies using self-reported measures have backed these findings [18,19]. One retrospective study found that half of the patients on opioids were able to stop, aided by the use of medical cannabis [20], and two studies reported that surveyed respondents used cannabis as a substitute for prescription opioids [21,22]. Other benefits provided by the addition of cannabis to opioids may be improved functional outcomes such as improved sleep [14] and quality of life [15,16,18], decreased side effects of medications [12,18], and reduced opioid tolerance and dependence [11].

Despite the aforementioned studies, it is still not widely accepted that cannabis may help with the opioid epidemic; the lack of clinical trials is cited as a primary reason. The ability to conduct well-designed clinical trials is inhibited by cannabis’ schedule I designation, which has led to a catch-22 situation in which the studies needed to prove or disprove efficacy cannot be practically conducted. Until more gold-standard trial data and clinical practice guidelines are available, clinicians must continue to manage patients who may choose to add cannabis to their opioid regimen for chronic pain. The United States’ Center for Disease Control 2016 guidelines for prescribing opioids for chronic pain recommended against urine screening for tetrahydrocannabinol (THC) in opioid-using patients with chronic pain due to uncertain clinical implications of a positive test result [23]. Unfortunately, many patients who have chosen to co-administer cannabis and opioids have been denied their prescriptions and/or discharged from medical care based on the presence of THC in the urine, potentially increasing the risk of obtaining and using opioid medications illegally. The purpose of this study was to examine the impact of medical cannabis on chronic pain patients who were using opioids. A secondary purpose was to assess participants’ ability to function and quality of life after using medical cannabis.

## Materials and methods

Study design and participants

This was a survey study of Integr8 Health, three affiliated cannabis medical practices in Falmouth Maine, Manchester Maine, and Burlington Massachusetts. A cannabis medical practice follows the Society of Cannabis Clinicians Practices Standards for Cannabis Approvals as defined by the Society of Cannabis Clinicians [24]. Integr8 Health had 16 healthcare providers across its three sites at the time of this survey.

On April 28, 2016, the listserve of Integr8 Health was emailed an invitation to participate in an anonymous convenience survey across its three practice sites. While there were over 18,000 patients within the three practice sites, the survey only asked for participants who had ever used opioids (including prescribed or illicit) to take an anonymous two-to-three-minute survey. Clicking on the survey link provided implied informed consent for the survey. A follow-up reminder email was sent on May 2, 2016. The study was open for five weeks and ended on June 3, 2016. Survey respondents received no compensation for participating.

Since we wanted to examine the effect of cannabis on chronic pain, we utilized the ICD-11 classification of pain, which assigns a benchmark of three months of persistent or recurrent pain [25]. We, therefore, excluded those patients who had not used prescribed opioid medications continuously for more than three months and those who did not use medical cannabis in combination with opioid use.

Survey instrument

**Figure 1:**
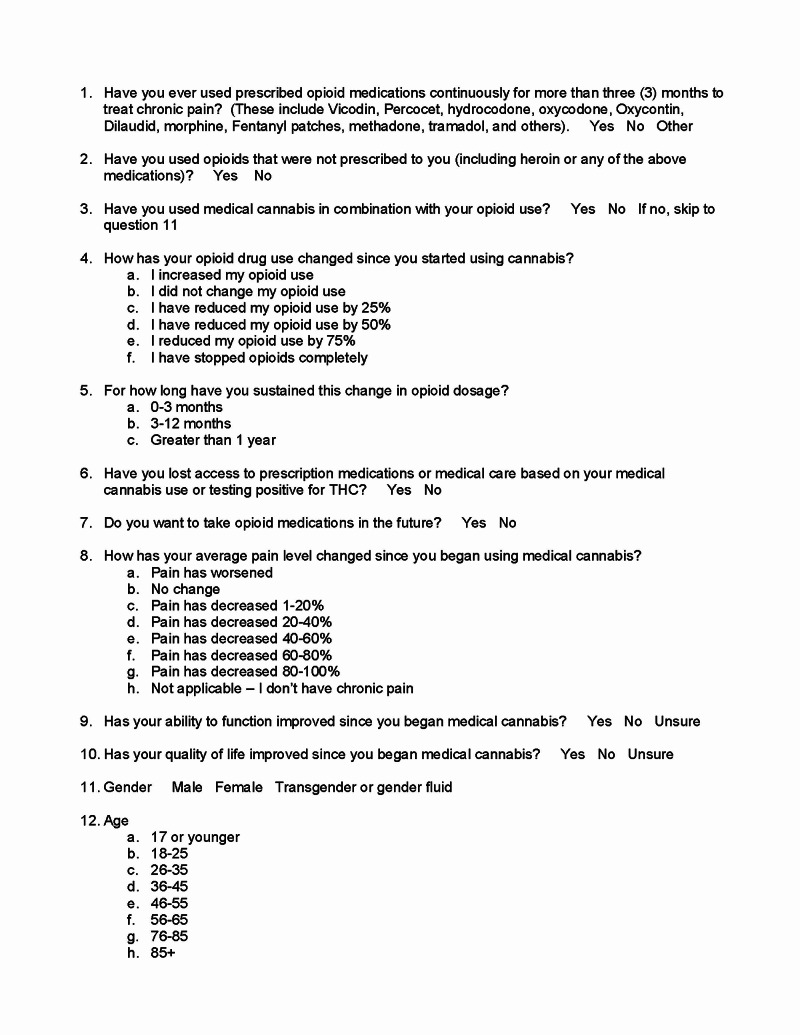
Survey questionnaire

The electronic survey was designed and written by one of the study authors (DS). He had observed that many patients in his clinics were taking opioids for chronic pain and seeking cannabis as an alternative method to address their pain. The survey consisted of 11 items: two demographic questions (gender, age range), two opioid usage questions assessing prescribed opioid usage for more than three months and opioid usage that was not prescribed, two opioid and cannabis questions assessing opioid usage since starting cannabis and how long the change lasted, three efficacy measures of cannabis use questions that assessed changes after using medical cannabis (average pain level, ability to function, quality of life) and two questions asking about the consequences of using medical cannabis (Figure 1).

The survey was a quality improvement initiative for the practice and utilized Google Forms. The study was reviewed by the Western Institutional Review Board and deemed to be exempt.

Data analysis

Frequency and percentage were reported for survey responses. Gender and age were used to predict responses to opioid usage questions, opioid plus cannabis use questions, efficacy measures of cannabis use questions, and consequences of using medical cannabis employing chi-square and binary logistic and multinomial logistic regressions. Age range categories were divided into younger (<46 years old), middle (46-55 years old), and older (>55) age groups. All analyses were performed using Statistical Package for Social Sciences (SPSS) Statistics for Windows, Version 25.0 (IBM Corp., Armonk, NY). P-value < 0.05 was considered statistically significant. Odds ratios with 95% confidence intervals are reported.

## Results

We received 1181 responses to the survey. We excluded 656 who reported they had not used prescribed opioid medications continuously for more than three months to treat chronic pain or had not used medical cannabis in combination with prescribed opioid use, leaving a final sample size of n=525 (Table 1).

**Table 1:** Study participant demographics and opioid usage

Demographics	N	%
Gender		
Male	289	55.8
Female	229	44.2
Age (in years)		
<18	1	0.2
18-25	17	3.2
26-35	59	11.2
36-45	108	20.6
46-55	165	31.4
56-65	137	26.1
66-75	36	6.9
>75	2	0.4
Opioid usage	Yes (%)	No (%)
Have you ever used prescribed opioid medications continuously for more than 3 months to treat chronic pain?	525 (45%)	641 (55%)
Have you used opioids that were not prescribed to you?	152 (29%)	373 (71%)

Results are reported in Table 2.

**Table 2:** Study participant medical cannabis use question responses *Percentages may not total 100% due to missing data or rounding.

Opioid + cannabis use questions		
How has your opioid drug use changed since you started using cannabis?	N	%*
No change	67	13.3
25% decrease	64	12.7
50% decrease	71	14.1
75% decrease	93	18.4
Stopped opioids	204	40.4
Increased opioids	6	1.2
For how long have you sustained this change in opioid dosage?		
0-3 months	50	10.9
3-12 months	109	23.8
> 12 months	299	65.3
Not applicable	67	
Efficacy measures of cannabis use questions		
How has your average pain level changed since you began using medical cannabis?	N	%
No change	43	8.6
1-20% decrease	89	17.8
20-40% decrease	114	22.8
40-60% decrease	121	24.2
60-80% decrease	97	19.4
80-100% decrease	23	4.6
Pain has worsened	13	2.6
Has your ability to function improved since you began medical cannabis?		
Yes	420	80.0
No	41	7.8
Unsure	64	12.2
Has your quality of life improved since you began medical cannabis?		
Yes	457	87.0
No	22	4.2
Unsure	46	8.8
Consequences of using cannabis questions		
Do you want to take opioid medications in the future?	N	%
Yes	136	26.5
No	323	62.8
If needed	55	10.7
Have you lost access to prescription medications or medical care based on your medical cannabis use or testing positive for THC?		
Yes	128	24.8
No	389	75.2

Age was significantly predictive of improvement in function since beginning medical cannabis, with the odds of the middle age group and older age group being 5.9 times (95% CI [1.947, 17.789], p < 0.01) and 5.5 times (95% CI [1.803, 16.736], p < 0.01) less likely to report improvement in function compared to younger individuals.

Age was significantly predictive of desire to continue using opioids, with the younger age group being 2.0 times more likely to report not wanting to use opioids again compared to the older age group (95% CI [1.284, 3.158], p < 0.01). There was a significant gender difference in the odds of reporting losing access to prescription medications or medical care as a result of medical cannabis use or testing positive for THC, with men being 1.8 times more likely than women to report losing access (95% CI [1.194, 2.759], p < 0.01).

There were significant age and gender differences with respect to the odds of using unprescribed opioids, with the middle age group and older age group being 4.6 times (95% CI [2.819, 7.628], p < 0.001) and 4.7 times (95% CI [2.890, 7.672], p < 0.001) less likely to use non-prescribed opioids compared to the younger group, and men being 1.8 times more likely to report use of non-prescribed opioids compared to women (95% CI [1.175, 2.697], p < 0.01). 

## Discussion

To our knowledge, this is one of the largest surveys of chronic pain patients who used opioids continuously for a minimum of three months and combined it with medical cannabis. Our results show a remarkable percentage of patients both reporting complete cessation of opioids and decreasing opioid usage by the addition of medical cannabis, with results lasting for over a year for the majority. We hypothesize these effects may be due to the reported synergistic decrease in pain that has been shown with adding cannabis to opioids [7,14,17]. Likely, as a result, the majority expressed not wanting opioids in the future, particularly those in the younger age group. Additional benefits of medical cannabis included improved ability to function and improved quality of life, especially for the younger age group.

One reason for our impressive results may be the focused protocol employed by the study sites [26], which recommends a small amount of oral cannabis (e.g. ratio of 1 mg of a balanced THC:cannabidiol (CBD) preparation) taken in conjunction with each opioid medication dose with small increments to titration, in a motivated patient population. There has never been a randomized controlled human trial examining how to use medical cannabis in combination with opioids and there is no established protocol that exists. Experts disagree on how to manage opioid prescriptions in patients with chronic pain who use cannabis, and many clinicians defer to the patient or dispensary agent on decisions regarding specific cannabis products and dosages [27]. In a recent observational study including 712 medical-only cannabis users with chronic pain, only 4.1% indicated that they had consulted with a medical professional about choosing cannabis products [28].

Furthermore, developing an individualized treatment plan may be complex to achieve given the multiple types and forms of cannabis in the marketplace and the need to consider adjunctive treatment of breakthrough symptoms. In the same medical-only cohort, 91% of patients used two or more delivery methods for cannabis, and 65.9% used three or more. The absence of a clinical protocol or medical management of the patients’ cannabis use patterns may explain the failure in other studies that purportedly show cannabis has no effect on opioid use [29]. Our results may be distinct due to the supportive treatment environment and follow-up provided by the medical cannabis clinics that worked to specifically help patients achieve their goals, which frequently include reduction of opioid medications.

Our results are consistent with a growing body of similar studies. One study of 176 treatment-resistant chronic pain patients found that pain and functional outcomes improved with medical cannabis and opioid use fell by 44% [14]. In a retrospective chart review study of 61 patients with chronic back pain who were taking prescription opioids and were using medical cannabis, 50.8% were able to stop opioid usage, which took a median of 6.4 years, while 31% of those who did not stop opioids were able to reduce usage [20]. A survey study of 244 chronic pain patients reported a 64% decrease in opioid use with medical cannabis [18]. In a survey of medical cannabis patients of whom 841 used opioid pain medication within the past six months, 74% strongly agreed and 23% agreed that cannabis allowed them to decrease their opioid usage, though just 61% used opioids with cannabis [19]. A study of 600 chronic pain patients taking an average daily morphine equivalent dose of 120 mg who were given medical cannabis and put on an opioid tapering plan found that after six months, 26% stopped using opioids and 55% reduced their opioid dose by 30% on average [30]. 

Limitations

Survey responses were taken from three practice sites in the northeast region of the country and therefore results are not necessarily generalizable to other regions of the country. The patient population served by these private-pay cannabis specialty clinics likely has selection bias with higher motivation to use cannabis as effectively as possible to achieve their goals, which often include substituting cannabis for opioids and other medications. As a result, there may be more positive results in this population compared to the general population. The survey was a convenience sample and results are subject to participation bias. Even though it was an anonymous survey, participants may have been hesitant to answer questions honestly due to the sensitive nature of the subject matter. We only included patients who reportedly had been using opioids continuously for more than three months, which we agreed defined those with sufficient and long enough pain to be considered chronic [25]. We did not determine exact opioid dosages or lengths of time taking opioids so cannot make quantitative assessments on their usage or duration. We did not define chronic pain or assess what chronic pain meant and did not determine the source or category of nociceptive, neuropathic, or other pain.

The response rate may have been affected by the need to understand the technology of taking an online survey. Potential participants may have been lost because it required access to the internet, an e-mail address and basic computer skills. Since this was a survey study, we had to rely on the memory and reported estimations of the participants. We could not verify actual changes in pain level, ability to function, or quality of life.

## Conclusions

We believe our results lend further support that medical cannabis provided in a standardized protocol can lead to decreased pain and opioid usage, improved function, and quality of life measures, and even complete cessation of opioids in patients with chronic pain treated by opioids. The younger age group showed more improvement in function, a greater likelihood of using unprescribed opioids, and less desire to use opioids again compared to their older counterparts. Our results are limited to those who sought care in a medical cannabis practice so maybe skewed to those willing to accept alternative approaches to pain management. Our study data relied on a questionnaire, perhaps the only practical way to obtain such information given the restrictive nature of obtaining cannabis for research purposes. Nonetheless, our results support the concept that medical cannabis may mitigate pain in those already taking opioids and help to diminish or even discontinue opioid usage. We hope these study results provide more impetus for further study in the form of randomized controlled trials if federal laws could be altered to support such studies.

## References

[1] Substance Abuse and Mental Health Services Administration (2019). Key substance use and mental health indicators in the United States: results from the 2018 National Survey on Drug Use and Health (HHS Publication No. PEP19-5068, NSDUH Series H-54). https://www.samhsa.gov/data/.

[2] Scholl L, Seth P, Kariisa M, Wilson N, Baldwin G (2019). Drug and opioid-involved overdose deaths—United States, 2013-2017. MMWR Morb Mortal Wkly Rep.

[3] Manzanares J, Corchero J, Romero J, Fernández-Ruiz JJ, Ramos JA, Fuentes JA (1999). Pharmacological and biochemical interactions between opioids and cannabinoids. Trends Pharmacol Sci.

[4] Cichewicz DL (2004). Synergistic interactions between cannabinoid and opioid analgesics. Life Sci.

[5] Russo EB, Hohmann AG (2013). Role of cannabinoids in pain management. Comprehensive Treatment of Chronic Pain by Medical, Interventional, and Integrative Approaches.

[6] Scavone JL, Sterling RC, Van Bockstaele EJ (2013). Cannabinoid and opioid interactions: implications for opiate dependence and withdrawal. Neuroscience.

[7] Nielsen S, Sabioni P, Trigo JM (2017). Opioid-sparing effect of cannabinoids: a systematic review and meta-analysis. Neuropsychopharmacology.

[8] Roberts JD, Gennings C, Shih M (2006). Synergistic affective analgesic interaction between delta-9-tetrahydrocannabinol and morphine. Eur J Pharmacol.

[9] Cooper ZD, Bedi G, Ramesh D, Balter R, Comer SD, Haney M (2018). Impact of co-administration of oxycodone and smoked cannabis on analgesia and abuse liability. Neuropsychopharmacology.

[10] Welch SP, Stevens DL (1992). Antinociceptive activity of intrathecally administered cannabinoids alone, and in combination with morphine, in mice. J Pharmacol Exp Ther.

[11] Cichewicz DL, Welch SP (2003). Modulation of oral morphine antinociceptive tolerance and naloxone-precipitated withdrawal signs by oral delta 9-tetrahydrocannabinol. J Pharmacol Exp Ther.

[12] Cichewicz DL, McCarthy EA (2003). Antinociceptive synergy between delta(9)-tetrahydrocannabinol and opioids after oral administration. J Pharmacol Exp Ther.

[13] Lynch ME, Clark AJ (2003). Cannabis reduces opioid dose in the treatment of chronic non-cancer pain. J Pain Symptom Manage.

[14] Haroutounian S, Ratz Y, Ginosar Y, Furmanov K, Saifi F, Meidan R, Davidson E (2016). The effect of medicinal cannabis on pain and quality-of-life outcomes in chronic pain: a prospective open-label study. Clin J Pain.

[15] Gruber SA, Sagar KA, Dahlgren MK (2018). The grass might be greener: medical marijuana patients exhibit altered brain activity and improved executive function after 3 months of treatment. Front Pharmacol.

[16] Bellnier T, Brown GW, Ortega TR (2018). Preliminary evaluation of the efficacy, safety, and costs associated with the treatment of chronic pain with medical cannabis. Ment Health Clin.

[17] Abrams DI, Couey P, Shade SB, Kelly ME, Benowitz NL (2011). Cannabinoid-opioid interaction in chronic pain. Clin Pharmacol Ther.

[18] Boehnke KF, Litinas E, Clauw DJ (2016). Medical cannabis use is associated with decreased opiate medication use in a retrospective cross-sectional survey of patients with chronic pain. J Pain.

[19] Reiman A, Welty M, Solomon P (2017). Cannabis as a substitute for opioid-based pain medication: patient self-report. Cannabis Cannabinoid Res.

[20] Takakuwa KM, Hergenrather JY, Shofer FS, Schears RM (2020). The impact of medical cannabis on intermittent and chronic opioid users with back pain: how cannabis diminished prescription opioid usage. Cannabis Cannabinoid Res.

[21] Lucas P, Walsh Z (2017). Medical cannabis access, use, and substitution for prescription opioids and other substances: a survey of authorized medical cannabis patients. Int J Drug Policy.

[22] Corroon JM Jr, Mischley LK, Sexton M (2017). Cannabis as a substitute for prescription drugs - a cross-sectional study. J Pain Res.

[23] Dowell D, Haegerich TM, Chou R (2016). CDC guideline for prescribing opioids for chronic pain - United States, 2016. JAMA.

[24] (2019). The Society of Cannabis Clinicians practice standards for cannabis approvals. https://cannabisclinicians.org/wp-content/uploads/2018/01/SCC-Standards-of-Practice.pdf.

[25] Treede RD, Rief W, Barke A (2015). A classification of chronic pain for ICD-11. Pain.

[26] Sulak D (2020). Medical cannabis opioid guide: how to use cannabis to reduce and replace opioid medications. http://healer.com/wp-content/uploads/2018/04/Healer-Medical-Cannabis-Opioid-Guide.pdf.

[27] Starrels JL, Young SR, Azari SS (2020). Disagreement and uncertainty among experts about how to respond to marijuana use in patients on long-term opioids for chronic pain: results of a Delphi study. Pain Med.

[28] Boehnke KF, Scott JR, Litinas E, Sisley S, Clauw DJ, Goesling J, Williams DA (2019). Cannabis use preferences and decision-making among a cross-sectional cohort of medical cannabis patients with chronic pain. J Pain.

[29] Campbell G, Hall WD, Peacock A (2018). Effect of cannabis use in people with chronic non-cancer pain prescribed opioids: findings from a 4-year prospective cohort study. Lancet Public Health.

[30] Rod K (2019). A pilot study of medical cannabis - opioid reduction program. Am J Psychiatry Neurosci.

